# A Generous Contribution to the Museum of the Dental Department of the University of Maryland

**Published:** 1883-01

**Authors:** 


*A Generous Contribution to the Museum of the Dental Department of the University of Maryland.*—Dr Genese who has been engaged in the practice of Dentistry for a number of years past in France and England, and who is a graduate of the Dental Department of the University of Pennsylvania, has presented to the Museum of the Dental Department of the University of Maryland a large number of valuable and interesting specimens, both pathological aud mechanical. Among the former are a number of skulls, maxillæ, abnormally shaped teeth, etc. etc; and among the latter artificial teeth carved from ivory and bone and mounted upon different bases, with many unique forms of teeth in use at an early period in the history of dentistry.

The Museum of the Dental Department of the University of Maryland is rapidly increasing, hundreds of valuable specimens having been presented since its organization, until at the present time it will compare favorably, in the value of the specimens, with Museums of much older institutions. Indeed, success appears to have followed every effort to make this Dental Department a true exponent of dental teaching: and the present session affords an evidence of the appreciation of the profession for increased facilities and advantages in the acquirement of dental knowledge.

---

In the December number of this JOURNAL some extracts from a paper on Dental Colleges, by Dr. A. Berry, were inadvertently ascribed to Dr. Watt, and as an act of justice to the former we make this acknowledgement.

